# Comparison of different drying technologies for green tea: Changes in color, non-volatile and volatile compounds

**DOI:** 10.1016/j.fochx.2024.101935

**Published:** 2024-10-28

**Authors:** Nannan Li, Zhengying Yao, Jingming Ning, Lijun Sun, Qunying Lin, Xiaoyan Zhu, Cuihong Li, Xiaohe Zheng, Jinghong Jin

**Affiliations:** a China CO-OP Nanjing Institute for Comprehensive Utilization of Wild Plants, Nanjing, Jiangsu Province 211111, China; b School of Tea and Food Science and Technology, Anhui Agricultural University, Hefei, Anhui Province 230036, China; c Tianfang Tea Industry Co., Ltd, Shitai, Anhui Province 245100, China

**Keywords:** Green tea, Vacuum freeze-microwave drying, Tea polyphenol, Caffeine, Color difference, Gas chromatography-ion mobility spectrometry (GC-IMS), Volatile component

## Abstract

Drying technology plays a pivotal role in tea processing. Herein, the differences in color, non-volatile, and volatile components of green tea under various drying methods were investigated. The results indicated that vacuum freeze-microwave increased the L* and b* values, and decreased the a* values of tea leaves. Moreover, vacuum freeze-microwave drying resulted in higher polyphenol content than the other three drying methods although there was no significant difference. A total of 43 volatile compounds were identified. Of these, 2-propanone, ethanol(D), ethanol(M), ethyl acetate(M), 2-methyl-1-butanol, and 2-methylthiophene were found to play an important role in the above discrimination (VIP >1.5). Dry extraction showed a higher content of volatile components than wet extraction. Regardless of the extraction conditions, vacuum freeze-microwave drying exhibited a stronger signal intensity and more volatile components than other drying methods. This study provides a reference for analyzing the quality differences of green tea by different drying methods.

## Highlights

• Vacuum freeze-microwave increased the L* and b* values, and decreased the a* values of green tea leaves.
• FMDT exhibits a higher polyphenol content than the other three drying methods.
• FMDT showed pronounced signal intensity and more volatile components.
• Dry extraction contains a higher content of volatile components.

## 1 Introduction

Tea (*Camellia sinensis*) is one of the top three non-alcoholic beverages consumed worldwide. It is associated with some health benefits, including antioxidants, inflammation, anti-aging action, anti-cancer activity, and prevention of hypertension and cardiovascular disease(Adhikary et al., 2023; da Silva Pinto, 2013; Liao et al., 2020). Green tea, one of China's most produced and consumed varieties of tea, has attracted significant interest due to its distinctive quality characteristics, including aroma, flavor, color, and tea soup clarity of the leaves(Liu, Shen, et al., 2023). The primary processes of green tea production involve fixation, rolling, and drying(Adhikary et al., 2023). As a crucial stage in the processing of green tea, drying plays a pivotal role in promoting flavor formation during the processing step for teas, while also extending the shelf life of the tea by reducing its water content(Tu et al., 2023) and inhibiting enzymatic activity(Kumar et al., 2023). Researchers are primarily concerned with the impact of conventional drying methods (hot-air drying, heat-conduction drying, and air drying) on tea color, aroma types, microorganisms, and volatile metabolites(Tu et al., 2023). Nevertheless, with the growing demand for tea production, conventional drying methods are no longer advantageous in improving tea leaves' production efficiency and product quality. Consequently, it is imperative to integrate advanced drying technologies into the production process.

At present, the tea drying process has been enhanced by the application of various drying techniques, including far-infrared drying(Qu et al., 2019), microwave drying(Dong et al., 2011), vacuum-freeze drying(Yang et al., 2024), and vacuum-microwave drying(Liu, Liu, et al., 2023). Moreover, the impact of drying on the chemical composition, sensory characteristics, and volatile compounds in various tea types has been reported(Tu et al., 2023; Wang, Ouyang, et al., 2022; Zhou et al., 2023). For example, Qu et al. (2019) reported that color, taste, phenolic compounds, and volatile compounds were sensitive to drying methods, among which microwave drying was found to be particularly effective in enhancing the polyphenol and theaflavin content of black tea, while also promoting the production of more volatile compounds(Qu et al., 2019). Yang et al. (2024) compared the influence of the three drying methods (traditional oven or air-drying, vacuum freeze drying) on the aroma, taste, and antioxidant activity of flower tea. The findings revealed that freeze drying was more effective in maintaining the color of the flowers and enhancing the taste and antioxidant activity of flower tea(Yang et al., 2024). However, the effects of vacuum freezing combined with microwave drying on the color, nonvolatile, and volatile components of green tea and their contributions remain poorly understood.

Besides, aroma is a crucial indicator of tea quality and is susceptible to external extraction conditions(Zhou et al., 2023). The volatile components of tea extracted by the dry method are predominantly volatiles on the surface of tea samples after heating. In contrast, the participation of a solvent allows for the full stretching of tea tissues, enabling the collection of soluble components in tea after dissolution and subsequent volatilization. Therefore, the content and composition of volatile components extracted by the wet method differ from those extracted by the dry method. It is necessary to investigate the impact of wet and dry extraction on the volatile components of tea dried by different methods, as well as to develop an effective aroma determination method.

Gas chromatography-ion mobility spectrometry (GC-IMS) is an emerging analytical technology that combines the high separation capacity of gas chromatography (GC) with the rapid response time of ion mobility spectrometry (IMS)(Zheng et al., 2023). With the advantages of ultrahigh sensitivity, lack of sample pretreatment requirement, operation under ambient pressures and temperatures, intuitive data visualization, excellent resolution, and rapid detection(Gu et al., 2021; Guo et al., 2022; Li et al., 2023; Xiao et al., 2022; Xu, Yao, et al., 2024). GC-IMS has been employed in the tea leaves area to identify the origin (Xiao et al., 2022; Zheng et al., 2023) and harvest seasons of tea(Liu et al., 2021), and to monitor changes in flavor substances during tea processing(Xie et al., 2023; Yang, Qian, et al., 2022) or storage(Rong et al., 2023; Xu, Zhang, et al., 2024). Nevertheless, only a limited number of studies have investigated the impact of distinct drying techniques on the aroma characteristics of green tea by GC-IMS.

To gain a more comprehensive understanding of the influence of different drying methods on the color, nonvolatile, and volatile components of green tea, this study selected green tea with different drying methods as samples. This study aimed to compare the differences in color, nonvolatile, and volatile components of green tea between the four drying patterns (hot-air drying, vacuum freeze-drying, microwave-drying, and vacuum freeze-microwave drying). Subsequently, the volatile components of tea were subjected to enrichment under different extraction conditions, after which its aroma characteristics were determined and analyzed by GC-IMS. The research offers a basis for the analysis of the quality differences in green tea resulting from different drying methods.

## 2 Materials and methods

### 2.1 Materials

The green tea tender leaves were harvested from the tea plantation at Chizhou, Anhui, China, in April 2022. Purified water was purchased from Hangzhou Wahaha Group Co., Ltd. (Hangzhou, China). Gallic acid was acquired from Beijing Biosynthesis Biotechnology Co. Ltd. (Beijing, China). Caffeine standard was obtained from Sigma-Aldrich (St-Louis, MO, USA). Chromatography-grade methanol was bought from Changshu Hongsheng Fine Chemical Co., Ltd. (Changshu, China).

### 2.2 Sample preparation

The green tea was subjected to four drying methods: hot-air drying tea (HDT), vacuum freeze-drying tea (FDT), vacuum microwave-drying tea (MDT), vacuum freeze-microwave drying tea (FMDT). Selected green tea has 2–3 tender leaves per bud with no obvious signs of damage or disease. Afterward, the sample was spreading, fixed, and rolling. The drying process and the final moisture content of green tea as illustrated in Table 1. For HDT, the samples were initially hot air drying at 100 °C for 30 min, followed by drying at 90 °C for 30 min, and then drying at 80 °C for 40 min. The FDT samples were pre-frozen at −40 °C and then dried for 20 h, where vacuum pressure at −90 ± 5 KPa, electric heating temperature at 30 ± 2 °C, freeze drying at −35 ± 5 °C. The MDT experiment was conducted with a vacuum microwave heating system. Before drying, the microwave output power was adjusted to 500 W, and the tea leaves were first dried at 30 °C for 1 h, then raised to 55 °C and continued drying for 2 h. FMDT was performed using a vacuum freeze-microwave heating system, the samples were freeze-dried at −35 ± 5 °C for 5 h, and then dried at 55 °C for 1 h under the microwave output power of 500 W.

**Table 1:** Processing conditions applied to tea leaves and drying methods required to obtain the final moisture content.

Samples	Drying temperature (°C)	Drying time (min)	Moisture content (%)
HDT	100	30	5.12 ± 0.21
	90	30	
	80	40	
FDT	−35	1200	2.46 ± 0.17
MDT	30	60	7.33 ± 0.16
	55	120	
FMDT	−35	300	5.33 ± 0.11
	55	60	

Note: HDT, hot-air drying tea; FDT, vacuum freeze-drying tea; MDT, vacuum microwave-drying tea; FMDT, vacuum freeze-microwave drying tea.

### 2.3 Color difference analysis of green tea

The color of tea leaves and tea infusions were measured using a CM-5 colorimeter (Konica Minolta, Japan) using a D65 light source. The color of tea leaves and tea infusions of HDT, FDT, MDT, and FMDT were described using the CIE L*a*b* system. L* represents lightness (0–100; black to white), a* represents redness (− to +; green to red), and b* represents yellowness (− to +; blue to yellow). Each sample was conducted in triplicate and distilled water was used as the normal control.

### 2.4 Tea polyphenols analysis

Following the GB/T 8313–2018 standard, the tea polyphenol content was determined by UV spectrophotometry. Specifically, 0.2 g of tea powder was combined with 5 mL of 70 % methanol in a water bath for 10 min, stirring every 5 min. After cooling to room temperature, the mixture was centrifuged at 3500 r/min for 10 min. The supernatant was then transferred to a 10 mL volumetric flask. Subsequently, the remaining residue was re-extracted by adding 5 mL of 70 % aqueous methanol, and the aforementioned procedure was repeated. The extraction solution was fixed and filtered through a 0.45 μm water membrane. Then, 1 mL of the filtrate was diluted to 100 mL with water, and 1 mL of the diluent was mixed with 5 mL of 10 % folinol reagent for 5 min, after which 4 mL of 7.5 % sodium carbonate solution was added and kept at room temperature for 60 min. The absorbance value was measured at a wavelength of 765 nm. The tea polyphenol content was calculated using the following formula:

$$c_{\mathrm{tp}}=\frac{\left(A-A_{0}\right)\times V\times d\times 100}{{\mathrm{SLOPE}}_{\mathrm{Std}}\times \omega \times 10^{6}\times m}$$

In this equation, A_0_ and A represent the absorbances of the blank and the sample, respectively; C_TP_ indicates the content of tea polyphenol; m means the weight of the sample; V is the volume of sample extract; d is the dilution factor; SLOPE_std_ is the slope of the gallic acid standard curve; ω is the dry matter content of the sample.

### 2.5 Caffeine analysis

The determination of caffeine content was conducted via the HPLC method (GB/T 8312–2013). Accurately weighed 0.2 g of tea powder and 0.9 g of magnesium oxide were dissolved in 60 mL of boiling water and extracted in a water bath for 20 min. The filtered tea infusion was collected in a 100 mL volumetric flask, cooled to room temperature, and made up to volume with distilled water. Subsequently, the tea infusion was then filtered through a 0.45 μm filter membrane. Subsequently, the 15 μL filtrate was injected into the HPLC apparatus, and a standard curve was drawn using a caffeine standard for chromatographic determination.

### 2.6 Water extract analysis

The water extract content was analyzed according to the method of GB/T 8305–2013. Tea powder (1 g) was extracted with 150 mL of boiled distilled water in a boiling water bath for 45 min. After vacuum filtering with filter paper, the tea residue was washed with approximately 150 mL of boiling distilled water. Finally, the tea residue was dried at 120 °C. Upon completion of the drying process, the sample was removed immediately, cooled to room temperature, and weighed.

### 2.7 Gas chromatography-ion mobility spectrometry analysis

A GC-IMS system (Flavourspec®, G.A.S, Dortmund, Germany) was used for volatile analysis. The system was equipped with an autosampler unit (CTC Analytics AG, Zwingen, Switzerland) and an MXT capillary column (15 m × 0.53 mm × 1 μm, Restek, PA, USA). Sample analysis was performed according to the methodology described by Yang, Chen, et al. (2022) with some slight modifications(Yang, Chen, et al., 2022). The tea powder (2 g) was weighed into 20 mL headspace vials and directly incubated at 80 °C for 15 min with a stirring speed of 500 rpm to obtain the dry extraction sample. The tea leaves (2 g) were soaked in boiling water for 3 min, and then incubated at 80 °C at a stirring speed of 500 rpm for 15 min to obtain the wet extraction sample. Then 500 μL of the headspace gas was injected into the inlet at 85 °C with a gastight syringe. Column and drift tube temperatures were maintained at 60 °C and 45 °C, respectively. Nitrogen (purity of 99.99 %) was used as the carrier gas. Carrier gas flow was programmed as follows: a flow rate of 2 mL/min was maintained for 2 min, after which it was linearly increased to 100 mL/min for 10–20 min and held for 10 min. All analyses were performed in triplicate.

The volatile compounds were identified by comparing their retention time and drift time with the NIST2004 and built-in IMS database obtained from G.A.S (Dortmund, Germany). The intensity of volatile compounds was analyzed based on the peak area of selected signal peaks using Gallery Plot analysis (v.1.0.3, G.A.S.). A two-dimensional top view was obtained using the Reporter plug-in of the GC-IMS, and the fingerprint profiles were generated using the Gallery Plot plug-in.

### 2.8 Statistical analysis

All experimental samples were prepared in triplicate and the data were listed as means ± standard deviation. One-way ANOVA was performed using SPSS 22.0, and Duncan's method was employed to evaluate the significant level of *P* < 0.05. Orthogonal partial least squares discriminant analysis (OPLS-DA) was performed to explore the differences during the different drying methods and extraction conditions, using SIMCA 14.1 software (Umetrics, Umea, Sweden). The pie chart was generated using Microsoft Excel 2021 (Microsoft Corporation, Redmond, WA).

## 3 Results and discussion

### 3.1 Color analysis of tea samples

**Fig. 1:**
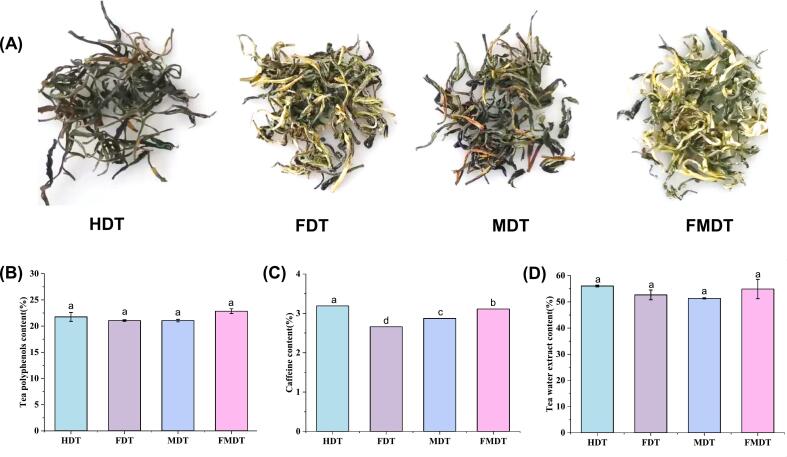
Changes of color, tea polyphenol, caffeine, and water extract content in green tea samples from different drying methods (A) Color differences; (B) Polyphenol; (C) Caffeine; (D) Water extract. HDT, hot-air drying; FDT, vacuum freeze-drying tea; MDT, vacuum microwave-drying tea; FMDT, vacuum freeze-microwave drying tea. (For interpretation of the references to color in this figure legend, the reader is referred to the web version of this article.)

Color is a crucial quality trait influencing consumer appetite, acceptance, and commodity value(Wang et al., 2023). Fig. 1 presents images of tea leaves subjected to four distinct drying methods. Following vacuum freezing and combined microwave drying, the color of the tea leaves becomes brighter. In contrast, the tea leaves dried by hot air and microwave are observed to have a fine, curled appearance and a dark green coloration. Generally, the drying rate was initially rapid in the hot-air drying process, but subsequently declined sharply. The rising hot air temperature was unable to enhance the drying rate in the final stage, yet it resulted in the quality of the tea. Similarly, under microwave conditions, the continuous provision of microwave energy results in a higher absorption rate, which in turn leads to a higher temperature and water transfer rate in the sample undergoing drying. This process causes the decomposition or oxidation of components such as chlorophyll and tea polyphenols, which results in the browning of the tea. Nevertheless, in the vacuum freeze-microwave drying process, vacuum freeze can effectively remove the majority of the water in the sample. Moreover, the higher vacuum pressures accelerate the drying speed of the tea leaves and reduce the average temperature of drying, which is conducive to the preservation of the color of the tea leaves.

In Table 2, we further determined their corresponding L*, a*, and b* values to assess the factors contributing to tea leaves color in greater detail. The measured L* values ranged from 31.85 to 42.73, indicating a significant variance in luminosity among the different dried tea leaves. Meanwhile, FDT and FMDT exhibited significantly lower a* values (ranging from −2.29 to −1.43) and substantially higher b* values (ranging from 19.74 to 23.25), which indicated a stronger greenness and a more pronounced yellowness in comparison with the HDT and MDT tea leaves. The color changes observed in the tea infusion were comparable to those observed in the tea leaves. That is, the L* and b* values of the tea infusion of FDT and FMDT were higher, and the a* values were lower, affirming a higher level of greenness and yellowness in their infusions.

**Table 2:** Color parameters of tea leaves and tea infusions according to different drying methods.

Tea leaves	L*	a*	b*
HDT	32.29 ± 0.28 ^*c*^	0.17 ± 0.04 ^*a*^	16.61 ± 0.11^*c*^
FDT	39.09 ± 0.36 ^*b*^	−1.43 ± 0.13 ^*c*^	19.74 ± 0.03^*b*^
MDT	31.85 ± 1.44 ^*c*^	−0.37 ± 0.24 ^*b*^	16.46 ± 1.13^*c*^
FMDT	42.73 ± 0.32 ^*a*^	−2.29 ± 0.09 ^*d*^	23.25 ± 0.61^*a*^
Tea infusions	L*	a*	b*
HDT	94.66 ± 0.01 ^*d*^	−2.55 ± 0.03 ^*a*^	9.04 ± 0.16 ^*c*^
FDT	95.07 ± 0.01 ^*a*^	−3.78 ± 0.02 ^*d*^	10.37 ± 0.09 ^*a*^
MDT	94.74 ± 0.01 ^*c*^	−2.99 ± 0.01 ^*b*^	9.77 ± 0.05 ^*b*^
FMDT	94.81 ± 0.01 ^*b*^	−3.11 ± 0.01 ^*c*^	9.86 ± 0.04 ^*b*^

Different letters in the same column indicate significant differences (P < 0.05). HDT, hot-air drying tea; FDT, vacuum freeze-drying tea; MDT, vacuum microwave-drying tea; FMDT, vacuum freeze-microwave drying tea.

### 3.2 Major compounds analysis of tea samples

**Fig. 2:**
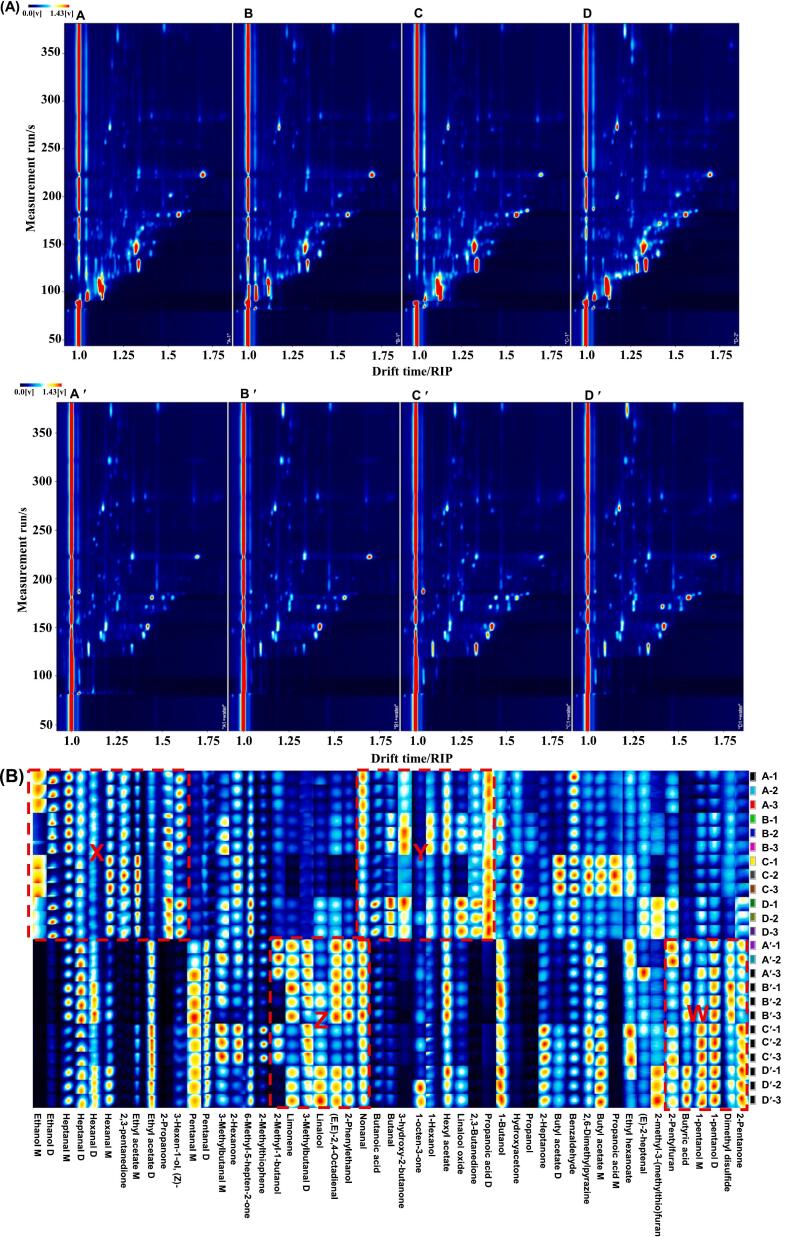
GC-IMS analysis of green tea from different drying methods. (A) Topographic plots; (B) Fingerprints of volatile compounds. A and A′, hot-air drying; B and B′, vacuum freeze-drying; C and C′, vacuum microwave-drying; D and D′, vacuum freeze-microwave drying. A-D, tea powder extraction at 80 °C; A′-D′, tea leaves soaked in 100 °C hot water and then extraction at 80 °C. (For interpretation of the references to color in this figure legend, the reader is referred to the web version of this article.)

Polyphenols are the main biochemical components in green tea that benefit human health(Ma et al., 2024). As shown in Fig. 2A, the content of tea polyphenols exhibited variability under disparate drying methods, though no significant difference was discerned in polyphenols between HDT, FDT, MDT, and FMDT. The FMDT exhibited a higher polyphenol content (22.85 %) than the HDT (21.75 %), FDT (21.05), and MDT (21.05). The changes in polyphenols are related to the variation of temperature and time in the four drying treatments. In general, high temperatures and long time increase the degradation of polyphenols. In the hot-air drying, the sample was dried at 80–100 °C, which resulted in partial damage of the polyphenols due to the relatively high temperature. In comparison to hot-air drying, the vacuum freeze-drying process required a relatively longer time, which accelerated the damage of polyphenols, leading to a relative reduction in the polyphenol content. Although microwave drying had a short drying time, the electromagnetic wave energy was considerable, resulting in a relative decrease in polyphenol content. The combination of vacuum freeze-drying and vacuum microwave-drying results in a higher polyphenol content than would be achieved through either process alone. This may be attributed to the fact that vacuum freeze-drying is an effective method for removing moisture from samples at low temperatures while also retaining thermosensitive substances in tea leaves. Furthermore, the application of a higher vacuum pressure can not only reduce the drying temperature of tea leaves, but also accelerate the drying rate. This is beneficial for the retention of tea polyphenols.

Caffeine is a significant taste component in tea, contributing to the bitterness of the tea brew (Wang et al., 2023). The drying process exerts a significant influence on the alteration of caffeine content. During the drying process, a proportion of the caffeine was lost due to sublimation, which reduced its content. The resulting trends are depicted in Fig. 2B. The caffeine content of tea samples subjected to different drying techniques ranged from 2.66 % to 3.19 %. The result was consistent with the findings of Donlao(Donlao & Ogawa, 2019), who reported that the caffeine content in the tea infusions exhibited variability, ranging from 1 to 7 g/100 g dried sample. A relative increase in the caffeine content was observed in HDT, followed by FMDT, FDT, and MDT, which may be related to both temperature and time during the drying process.

Water extracts are soluble substances in green tea and are composed of a variety of compounds, including polyphenolics, catechin, carbohydrates, amino acids, alkaloids, pigments, vitamins, and other trace elements(Kim et al., 2016). As shown in Fig. 2C, the water extract content showed an almost similar trend to tea polyphenols, although no significant differences were observed. Thus, the higher water extract could be partly because of higher polyphenolic content.

### 3.3 GC-IMS comparison analysis of volatile components in green tea with different drying methods and extraction conditions

GC-IMS is a powerful analytical technique for the separation and sensitive detection of volatile compounds(Feng et al., 2024; Wang et al., 2020). The volatile compounds of tea (e.g., black tea, white tea, and green tea) are significantly affected by different drying methods (Chen et al., 2023; Qu et al., 2019; Tu et al., 2023; Wang, Su, et al., 2022). To elucidate the changes of volatile compounds in green tea dried in different ways (including hot-air drying, vacuum freeze-drying, vacuum microwave-drying, and vacuum freeze-microwave drying), the samples were analyzed via GC-IMS.

**Fig. 3:**
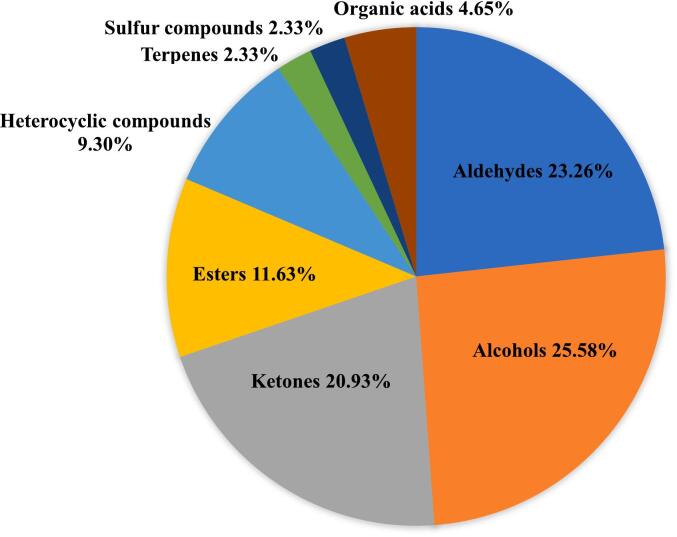
The relative percentages of compound numbers.

Differential two-dimensional (2D) topographic plots of the volatile components in the green tea are shown in Fig. 3. The Y-axis represents the gas chromatography retention time, and the X-axis represents the ion migration time. Given that minor alterations in capillary column temperature and gas flow can cause changes to retention time, it is necessary to normalize the reactive ion peak (RIP) to a value of 1. Each point on the right of the RIP corresponds to a volatile component. It can be observed that the majority of the signals were distributed within a retention time range of 100 to 300 s and a drift time of 1.0 to 1.75. The colors represent the concentration of each compound. The darker color (closer to the red) means a higher substance concentration, and the lighter color (closer to the blue background) means the lower the substance concentration. As shown in Fig. 3A, under identical extraction conditions, the signal distributions of numerous peaks in the samples were found to be analogous, whereas notable discrepancies were observed in the peak intensities of the volatile components across the various drying methods. However, regardless of the extraction conditions, the signal intensity of vacuum freeze-microwave drying green tea is stronger than that of green tea dried by the other three methods. Nevertheless, Zhou et al. compared the effects of three different drying methods (i.e. low-temperature drying, hot air drying, and sunlight drying) on the volatile metabolites of Ganpu Tea. The results demonstrated that sunlight-drying Ganpu Tea significantly increased the levels of volatile metabolites(Zhou et al., 2023). This research confirms the notion that the volatile component and content of tea were markedly affected by the drying methods employed.

In addition, the extraction conditions also affect the volatile components and content of the tea leaves. As illustrated in Fig. 3A, the tea powder extracted directly at 80 °C (A-D) exhibited darker colors and larger areas of the dots, indicating higher concentrations of the substance, compared to the tea leaves soaked in 100 °C hot water and then extracted at 80 °C (A′-D′). It was postulated that hot water immersion accelerates the release of volatiles, thereby interfering with the probe's ability to capture aroma compounds.

### 3.4 Fingerprint analysis of volatile compounds of green tea samples obtained from different drying methods and extraction conditions

Although the above two-dimensional plots illustrate the differences in volatile compound concentration under different drying methods and extraction conditions, it was difficult to make accurate judgments about specific volatile compounds. Hence, all the signal peaks were utilized for the subsequent fingerprint analysis. Each row represented the entire signal of one sample, and each column represented the content distribution of the same compound in different samples. Notably, the color of a fingerprint indicates the concentration of a volatile component, and a lighter color indicates a higher amount.

As shown in Fig. 3B, the volatile compounds in green tea from different drying methods or extraction conditions exhibit similarities. However, each drying method or extraction condition possesses its unique constituents. According to different extraction conditions, the fingerprints of volatile compounds could be divided into four regions (X, Y, Z, and W). Among them, regions X and Y represent the typical volatile compounds extracted from tea powder at 80 °C. For example, some volatile compounds including ethanol, 2,3-pentanedione, 2-propanone, 3-hydroxy-2-butanone, 2,3-butanedione, propanoic acid, hydroxyacetone, and propanol exhibited high concentrations for the A-D samples (tea powder extraction at 80 °C), whereas the A′-D′ samples (tea leaves are soaked in hot water at 100 °C and then extracted at 80 °C) lacked these compounds. Regarding Z and W regions associated with the other extraction method (tea leaves soaked in 100 °C hot water and then extracted at 80 °C), some volatile compounds including 2-methyl-1-butanol, limonene, 3-methyl butanal, linalool, (E, E)-2,4-octadienal, 2-phenyl ethanol; 2-pentyl furan, butyric acid, 1-pentanol, dimethyl disulfide, and 2-pentanone showed higher concentrations in the A′-D′ samples than in the A-D samples.

Compared with other conventional drying methods (e.g., hot-air drying, vacuum freeze-drying), vacuum microwave-drying and vacuum freeze-microwave drying increase the diversity of volatile compounds in green tea. The results indicated that microwaves may facilitate the polymerization and condensation of volatile substances, as well as the release of distinctive volatile compounds. Similarly, previous studies have demonstrated that microwave drying can augment the variety of volatile substances in Pu-erh tea (Wang, Su, et al., 2022).

### 3.5 Qualitative analysis of the volatile compounds of green tea

**Fig. 4:**
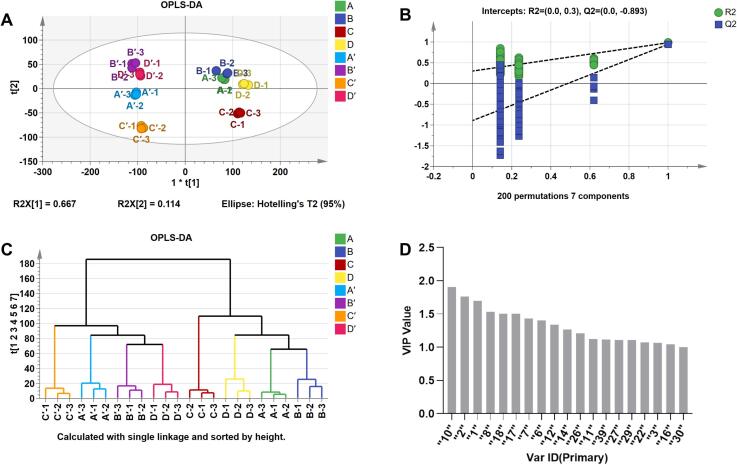
OPLS-DA results obtained from GC-IMS analysis under different drying methods and extraction conditions. (A) OPLS-DA scores plots (R2X = 0.988, R2Y = 0.969, Q2 = 0.913); (B) Cross-validation results with 200 times of calculations by using a permutation test (R2 = 0.3, Q2 = − 0.893); (C) HCA of the selected vector; (D) Volatiles ranked by VIP scores. A and A′, hot-air drying; B and B′, vacuum freeze-drying; C and C′, vacuum microwave-drying; D and D′, vacuum freeze-microwave drying. A-D, tea powder extraction at 80 °C; A′-D′, tea leaves soaked in 100 °C hot water and then extraction at 80 °C.

To qualitatively analyze the volatile components of green tea under different drying methods and extraction conditions, the fingerprints were employed to compare the retention time and drift time with those of the authentic reference compounds. Due to the differing concentrations of the volatile compounds, some volatile compounds produce multiple signals or spots (dimers or even trimers). In this study, a total of 43 typical volatile compounds (51 peaks) were identified from the topographic plots, and all the identified volatile compounds could be categorized into eight groups, including 11 alcohols, 10 aldehydes, 9 ketones, 5 esters, 4 heterocyclic compounds, 1 terpene, 1 sulfur-containing compound, and 2 organic acids (Table 3). Of these, alcohols were the most abundant (25.58 %), consistent with previous reports(Liu, Shen, et al., 2023; Qin et al., 2024), followed by aldehydes (23.26 %), ketones (20.93 %), and esters (11.63 %) (Fig. 4).

**Table 3:** The information of identified volatile compounds of green tea with different drying methods and extraction conditions by using GC-IMS.

No.	Compound	CAS#	Formula	MW	RI	Rt [sec]	Dt [RIPrel]
1	Ethanol(M)	C64175	C_2_H_6_O	46.1	445.5	103.487	1.1353
2	Ethanol(D)	C64175	C_2_H_6_O	46.1	428.9	101.366	1.0478
3	Heptanal(M)	C111717	C_7_H_14_O	114.2	886.5	223.701	1.6945
4	Heptanal(D)	C111717	C_7_H_14_O	114.2	892.6	226.766	1.3375
5	Hexanal(D)	C66251	C_6_H_12_O	100.2	793.2	183.632	1.2639
6	Hexanal(M)	C66251	C_6_H_12_O	100.2	787.3	181.473	1.5624
7	2,3-pentanedione	C600146	C_5_H_8_O_2_	100.1	678.3	147.518	1.3234
8	Ethyl acetate(M)	C141786	C_4_H_8_O_2_	88.1	606.5	130.532	1.3372
9	Ethyl acetate(D)	C141786	C_4_H_8_O_2_	88.1	614.7	132.275	1.0972
10	2-Propanone	C67641	C_3_H_6_O	58.1	509.4	112.675	1.121
11	3-Hexen-1-ol, (*Z*)-	C928961	C_6_H_12_O	100.2	839	201.903	1.5142
12	Pentanal(M)	C110623	C_5_H_10_O	86.1	696.8	152.526	1.4227
13	Pentanal(D)	C110623	C_5_H_10_O	86.1	699.2	153.198	1.1854
14	3-Methylbutanal(M)	C590863	C_5_H_10_O	86.1	651.8	140.824	1.4048
15	2-Hexanone	C591786	C_6_H_12_O	100.2	785.6	180.842	1.5057
16	6-Methyl-5-hepten-2-one	C110930	C_8_H_14_O	126.2	973.5	272.736	1.1758
17	2-Methylthiophene	C554143	C_5_H_6_S	98.2	804.2	187.807	1.042
18	2-Methyl-1-butanol	C137326	C_5_H_12_O	88.1	786.7	181.226	1.4565
19	Limonene	C138863	C_10_H_16_	136.2	1042.6	321.939	1.2101
20	3-Methylbutanal(D)	C590863	C_5_H_10_O	86.1	669	145.09	1.1631
21	Linalool	C78706	C_10_H_18_O	154.3	1103.3	374.411	1.2173
22	(E, E)-2,4-Octadienal	C30361285	C_8_H_12_O	124.2	1122.9	393.341	1.2649
23	2-Phenylethanol	C60128	C_8_H_10_O	122.2	1121.1	391.612	1.3116
24	Nonanal	C124196	C_9_H_18_O	142.2	1107.7	378.571	1.4769
25	Butanoic acid(M)	C107926	C_4_H_8_O2	88.1	837.2	201.118	1.1801
26	Butanal	C123728	C_4_H_8_O	72.1	587.1	126.523	1.2894
27	3-hydroxy-2-butanone	C513860	C_4_H_8_O_2_	88.1	730.1	162.249	1.333
28	1-octen-3-one	C4312996	C_8_H_14_O	126.2	987.4	281.816	1.6787
29	1-Hexanol	C111273	C_6_H_14_O	102.2	858	210.25	1.3274
30	Hexyl acetate	C142927	C_8_H_16_O_2_	144.2	994.1	286.362	1.4045
31	Linalool oxide	C60047178	C_10_H_18_O_2_	170.3	1087.9	360.17	1.2582
32	2,3-Butanedione	C431038	C_4_H_6_O_2_	86.1	536.2	117.091	1.1694
33	Propanoic acid	C79094	C_3_H_6_O_2_	74.1	650.8	140.56	1.2825
34	1-Butanol	C71363	C_4_H_10_O	74.1	640.3	138.056	1.1772
35	Hydroxyacetone	C116096	C_3_H_6_O_2_	74.1	640.4	138.08	1.2246
36	Propanol	C71238	C_3_H_8_O	60.1	550.2	119.538	1.243
37	2-Heptanone	C110430	C_7_H_14_O	114.2	876.6	218.892	1.2617
38	Butyl acetate(D)	C123864	C_6_H_12_O_2_	116.2	799.2	185.879	1.6196
39	Benzaldehyde	C100527	C_7_H_6_O	106.1	948.9	257.555	1.1496
40	2,6-Dimethylpyrazine	C108509	C_6_H_8_N_2_	108.1	909.4	235.399	1.1382
41	Butyl acetate(M)	C123864	C_6_H_12_O_2_	116.2	801.7	186.847	1.2379
42	Propanoic acid(M)	C79094	C_3_H_6_O_2_	74.1	721.9	159.761	1.104
43	Ethyl hexanoate	C123660	C_8_H_16_O_2_	144.2	986.7	281.344	1.3415
44	(E)-2-heptenal	C18829555	C_7_H_12_O	112.2	941.1	252.958	1.2563
45	2-methyl-3-(methylthio)furan	C63012975	C_6_H_8_OS	128.2	961.5	265.189	1.1063
46	2-Pentylfuran	C3777693	C_9_H_14_O	138.2	982	278.212	1.2544
47	Butyric acid(D)	C107926	C_4_H_8_O_2_	88.1	843.9	204.034	1.1536
48	1-Pentanol(M)	C71410	C_5_H_12_O	88.1	754.5	170.059	1.516
49	1-Pentanol(D)	C71410	C_5_H_12_O	88.1	750.5	168.742	1.2544
50	Dimethyl disulfide	C624920	C_2_H_6_S_2_	94.2	764.5	173.405	1.1284
51	2-Pentanone	C107879	C_5_H_10_O	86.1	695	152.032	1.3865

Note: MW, molecular mass; RI, relative retention index; Rt, retention time; Dt, relative migration time.

Alcohols typically exhibit floral-like aroma characteristics(Liu, Shen, et al., 2023). The floral-like aroma is considered a characteristic of high-quality tea leaves(Feng et al., 2020). 1-hexanol and linalool oxide were the main volatiles responsible for the floral-like aroma in green tea, while 3-methylbutanal contributes to producing the chestnut-like aroma(Liu, Shen, et al., 2023), which is also widely regarded as an important indicator of excellent green tea quality. The specific flavor of green tea is usually produced during high-temperature processing(Zhu et al., 2018).

### 3.6 The multivariate statistical analysis in different drying methods and extraction conditions

**Fig. 5:**
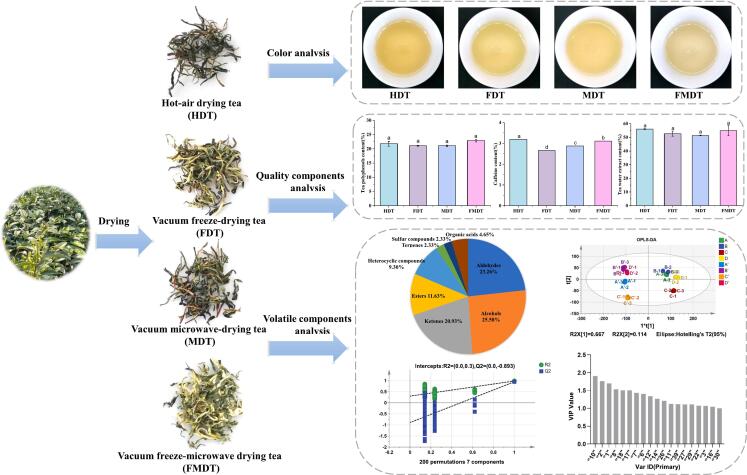
Effect of various drying methods on color, non-volatile and volatile components of green tea. (For interpretation of the references to color in this figure legend, the reader is referred to the web version of this article.)

The differentiation of green tea from different drying methods and extraction conditions can be effectively achieved using OPLS-DA, with 43 volatile substances as dependent variables and drying methods and extraction conditions as independent variables. The OPLS-DA model showed clear discrimination among the four drying methods and two extraction conditions of green tea, with each drying method and extraction condition exhibiting relatively concentrated distributions (Fig. 5A). R2X and R2Y represent the interpretation rates of the built model for the X and Y matrices, respectively, and Q2 represents the predictive power of the model. The closer these three metrics are to 1, the more stable and reliable the model. In the analysis, the value of the independent variable fit index (R2X) was determined to be 0.988, while the dependent variable fit index (R2Y) was determined to be 0.969. Additionally, the model prediction index (Q2) was calculated to be 0.913, indicating a good fit with high predictive accuracy.

To further investigate the robustness of the model, 200 permutation tests were performed. As shown in Fig. 5B, all the Q2 values on the left side were lower than the original point on the right side, and the intercept between the Q2 regression line and the Y-axis was less than 0, indicating that there was no overfitting phenomenon, and the OPLS-DA model verification was effective. The dendrogram of hierarchical clustering based on the OPLS-DA result showed the accurate clustering of two groups of tea samples with different drying methods and extraction conditions. As illustrated in Fig. 5C, the results of the hierarchical clustering analysis (HCA) tree structure were consistent with the OPLS-DA score plots, with the eight samples being separated into two main groups based on the two extraction conditions. Furthermore, four different drying methods were clearly distinguished in each group. Thus, the GC-IMS was able to rapidly distinguish different drying methods and extraction conditions of green tea, thereby facilitating the extraction and identification of tea aroma.

Variable Importance in Projection (VIP) was used to identify the significant volatile components that helped discriminate the two groups. The judging criteria with VIP > 1 were generally considered to play an important role in aroma quality. To better determine the contribution of various volatile substance components to the aroma of green tea, we analyzed the volatile components of tea leaves from different drying methods and extraction conditions and screened for the volatile components with VIP > 1. A total of 19 volatile compounds with VIP values greater than 1.0 were identified. Of these, 2-propanone (No. 10), ethanol(D) (No. 2), ethanol(M) (No. 1), ethyl acetate(M) (No. 8), 2-methyl-1-butanol (No. 18) and 2-methyl thiophene (No. 17) with VIP values greater than 1.5 were particularly noteworthy (Fig. 5D). These compounds are considered to be the main contributors to the differences in the aroma characteristics among the four drying treatments and two extraction conditions in green tea.

## 4 Conclusion

In summary, the drying methods showed remarkable effects on the color, nonvolatile, and volatile components of green tea (Fig. 5). In this study, FMDT exhibited significantly lower a* values, and higher L* and b* values, indicating brighter green hue and a more pronounced yellowish tint than the HDT and MDT tea leaves. Furthermore, the tea infusion of FDT and FMDT also demonstrated a comparable trend. The FMDT exhibited a higher polyphenol content than the HDT, FDT, and MDT. The water extract content exhibited a trend nearly identical to tea polyphenols, though no statistically significant differences were identified. The OPLS-DA model showed unambiguous differentiation among the four drying methods and two extraction conditions of green tea, with each drying method and extraction condition exhibiting a relatively concentrated distribution. The tea powder extracted directly at 80 °C (dry extraction) exhibited higher concentrations of the substance than the tea leaves that were soaked in 100 °C hot water and then extracted at 80 °C (wet extraction). A total of 43 typical volatile compounds (51 peaks) were identified, and all the identified volatile compounds could be classified into eight categories, mainly alcohols (11), aldehydes (10), ketones (9), esters (5), heterocyclic compounds (4), terpene (1), sulfur-containing compounds (1), and organic acids (2). Among them, alcohols were the most abundant with 23.26 % of the total, followed by aldehydes (23.26 %), ketones (20.93 %), and esters (11.63 %). Nineteen volatile compounds were found to play a crucial role in distinguishing the four drying methods and two extraction conditions of green tea. Of these, 2-propanone, ethanol(D), ethanol(M), ethyl acetate(M), 2-methyl-1-butanol, and 2-methylthiophene, which exhibited VIP values exceeding 1.5, were of particular importance. This study offers theoretical guidance for the directional processing and quality improvement of green tea and new insights into applying different drying methods in green tea production.

## CRediT authorship contribution statement

**Nannan Li:** Writing – original draft, Visualization, Formal analysis, Data curation. **Zhengying Yao:** Formal analysis, Data curation. **Jingming Ning:** Supervision, Resources, Methodology. **Lijun Sun:** Supervision, Methodology. **Qunying Lin:** Software, Methodology. **Xiaoyan Zhu:** Methodology, Formal analysis. **Cuihong Li:** Resources, Methodology, Conceptualization. **Xiaohe Zheng:** Resources, Conceptualization. **Jinghong Jin:** Writing – review & editing, Supervision, Project administration, Funding acquisition.

## Declaration of competing interest

The authors declare that they have no known competing financial interests or personal relationships that could have appeared to influence the work reported in this paper.

## Data Availability

Data will be made available on request.
